# QI: Improving Physical Healthcare Recording in a Mental Health Service for Homeless People – Working With KPI's

**DOI:** 10.1192/bjo.2022.298

**Published:** 2022-06-20

**Authors:** Hugh Hall, Michael Ward, Georgina Wicks, Katarzyna Sulej, Abena Agyapongmaa

**Affiliations:** 1 College of North West London, London, United Kingdom; 2 Chelsea and Westminster Hospital NHS Foundation Trust, London, United Kingdom

## Abstract

**Aims:**

As a mental health team for homeless people, we are aware of poor health outcomes for our patients. They face the double-hit of chronic serious mental illness (SMI) and homelessness, reducing life expectancy. As outlined in guidance, “secondary care team should maintain… monitoring service user's physical health”. We aimed to improve recorded annual physical health checks according to Trust Key Performance Indicators (KPI) for weight; hypertension; diabetes; cholesterol; and screening for smoking, drugs and alcohol on SystmOne (e-patient record) in Westminster's Joint Homelessness Team's (JHT) caseload, with target of 90% by December 2021 set by Central and North West London (CNWL) NHS Trust.

**Methods:**

Using monthly physical health KPI reports to target uncompleted annual health checks for JHT's 135 patients. PDSA cycles were used over a six-month period from July 2021 – January 2022.

Intervention 1: Using available GP data to pull across into our records, making use of existing information.

Intervention 2: Dedicated clinical session from FY2 doctor to assess patients with missing physical health checks.

Intervention 3: Specific teaching to whole MDT to increase awareness and uptake.

Intervention 4: Designed our own reporting to give real-time rather than monthly reporting.

Outcomes were measured from monthly Physical Health reports for the active caseload.

**Results:**

At baseline only 26.67% of patients had completed recorded health checks. Intervention 1 more than doubled our recordings to 54.17% over a 2-month period. Our second intervention further improved recorded physical health checks.

The third intervention increased our recorded physical health checks to 82.35% over a 2-month period. Notably, at the beginning of our project 7 out of 135 patients, had no engagement in physical health check monitoring, this reduced to 1 after intervention 3.

At the end of our fourth cycle, we had increased our recorded physical health checks to 83.93%.

Overall, results show an improvement of 57.26%, or a relative increase of 3.15 times the amount of recorded physical health checks over 6 months.

**Conclusion:**

As a result of incorporating dedicated clinical time, teaching and real-time use of data, we have improved our recorded physical health checks. There is room for improvement with 16% of patients still with incomplete health checks and approximately 10% of patients without blood tests. Some of this is due to accessibility and engagement difficulties for people with SMI and entrenched rough-sleeping, with ongoing work being done.

